# Applications of Biopolymers for Drugs and Probiotics Delivery

**DOI:** 10.3390/polym13162729

**Published:** 2021-08-15

**Authors:** Roxana Gheorghita, Liliana Anchidin-Norocel, Roxana Filip, Mihai Dimian, Mihai Covasa

**Affiliations:** 1 Department of Health and Human Development, Stefan cel Mare University of Suceava, 720229 Suceava, Romania; roxana.puscaselu@usm.ro (R.G.); liliana.norocel@usm.ro (L.A.-N.); 2 Integrated Center for Research, Development and Innovation in Advanced Materials, Nanotechnologies, and Distributed Systems for Fabrication and Control, Stefan cel Mare University of Suceava, 720229 Suceava, Romania; dimian@usm.ro; 3 Hipocrat Clinical Laboratory, 720003 Suceava, Romania; roxana.filip@usm.ro; 4 Department of Computers, Electronics and Automation, Stefan cel Mare University of Suceava, 720229 Suceava, Romania; 5 Department of Basic Medical Sciences, College of Osteopathic Medicine, Western University of Health Sciences, Pomona, CA 91766, USA

**Keywords:** drug, controlled release, polysaccharide, probiotics

## Abstract

Research regarding the use of biopolymers has been of great interest to scientists, the medical community, and the industry especially in recent years. Initially used for food applications, the special properties extended their use to the pharmaceutical and medical industries. The practical applications of natural drug encapsulation materials have emerged as a result of the benefits of the use of biopolymers as edible coatings and films in the food industry. This review highlights the use of polysaccharides in the pharmaceutical industries and as encapsulation materials for controlled drug delivery systems including probiotics, focusing on their development, various applications, and benefits. The paper provides evidence in support of research studying the use of biopolymers in the development of new drug delivery systems, explores the challenges and limitations in integrating polymer-derived materials with product delivery optimization, and examines the host biological/metabolic parameters that can be used in the development of new applications.

## 1. Introduction

Biopolymers are generated by living organisms [1,2,3,4,5] and are defined as biologically degradable polymers [6]. They represent possible materials for the replacement of synthetic plastics due to an increased interest in developing environmental sustainability [7]. Biopolymers have a structural backbone with carbon, oxygen, and nitrogen atoms which makes them easily biodegradable. Biodegradation breaks them down into carbon dioxide, water, humic matter (organic macromolecular material), biomass, and other natural substances; thus, these materials are naturally recycled through biological processes [3].

**Figure 1:**
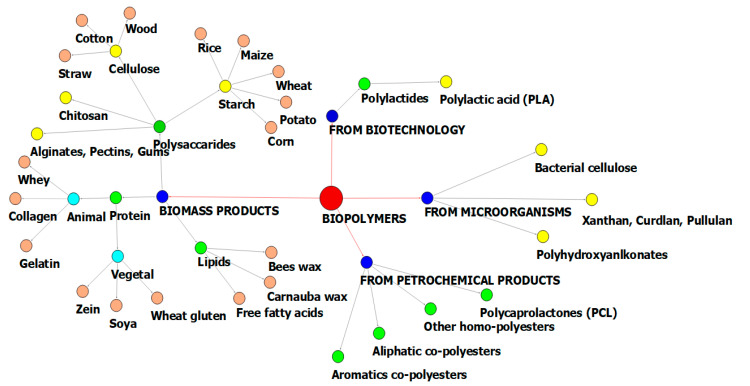
A social network graphical illustration of biopolymers classification.

A classification system based on their origin, synthesis and processing of different biodegradable polymers [8,9,10,11,12] is presented in Figure 1 in the form of a social network analysis. It divides the biopolymers in four major categories: extracted from biomass products (agrobiopolymers), from microorganisms, and from biotechnological and petrochemical products. Biopolymers from biomass products have diverse compounds such as polysaccharides (starches, celluloses, alginates, pectins, gums, and chitosan) [13]; proteins of animal origin (whey, collagen, and gelatin); proteins of vegetal origin (zein, soya, and wheat gluten) [14,15]; and lipids (bees wax, carnauba wax, and free fatty acids) [16,17]. Most biopolymers can be extracted from natural sources such as plants, animals, and microorganisms including algae and agro-wastes [18]. Agro-sources of biopolymers include bananas, maize, potatoes, tapioca, yams, rice, corn, wheat, cotton, sorghum, and barley [19,20], while animal sources are derived from cattle, pigs, and other products. Agro-waste-based sources include apple pomace [21], tomato pomace, pineapple [22], orange and lemon peels, wheat straw, rice husks [23,24], paper wastes, crops, wood, and green wastes, while the marine sources are sponges [25], corals, lobsters, fishes, and shrimps [26]. Biomaterials manufactured from these products are described as stretchy, soft, and gel-like, with many characteristics of both solids and fluids. It is known that biopolymers can be smart and flexible materials even in living organisms [6] because they have a structure that is constantly manipulated either in response to environmental changes or by enzymes throughout different stages of the organism’s lifecycle [27].

The biopolymer composites can be prepared by several methods such as extrusion, electrospinning, grafting, different types of molding [28], solvent casting, melt blending, intercalation, filament winding, phase separation [29], laser printing, and film stacking [5].

Currently, the manufactured design and optimization of biopolymers through mathematical models are very advantageous because they improve their physical, chemical, electrical, and mechanical properties in order to increase resistance in humid, warm, or cold storage conditions and for applications that require specific features [30].

Although significant work has been done on the use of a wide range of biopolymers, most of them have been based on polysaccharides due to their improved properties compared to other categories such as proteins or lipids. Thus, this review focuses on the ability of biopolymers to be used successfully in the pharmaceutical industry as encapsulating agents, particularly for delivery of drugs and probiotics. Specifically, the paper describes the use of alginate, chitosan, agar, starch, and cellulose by focusing on the properties and characteristics that make them suitable candidates for product delivery, offering advantages over the chemically derived polymers. The following will also be presented: the development of encapsulated substances based on biopolymers; challenges and limitations such as the encapsulation process, shelf life, controlled release of embedded drugs, protection, and viability of live strains; and the rate of release at different pH mediums of the gastrointestinal fluids.

## 2. Biopolymers vs. Conventional Synthetic Materials

Several studies have been conducted concerning the utilization of biopolymers with the aim of developing sustainable packaging materials. Although significant improvements have been made, there is still considerable debate over economic considerations, environmental concerns, and product packaging performance [31].

Living organisms produce a variety of polymers as a significant part of their morphological, cellular, and dry matter. These biopolymers play vital roles in the life cycle of organisms including the preservation and expression of genetic information, catalysis of reaction [32], energy or other nutrients, sensing of abiotic and biotic factors, protecting against the attack of other cells, storage of carbon, and negotiation of the adhesion to the surface of other organisms [4].

Biopolymers present important features such as biodegradability [33,34], biocompatibility [35], sustainability [36], bioresorbability [37], flexibility [38], antibacterial activity [6], renewability [39], and stability [2]. They are also less toxic [40], non-immunogenic [41], non-carcinogenic, non-thrombogenic, carbon neutral, and have the advantage of easy extraction [42]. These properties are directly influenced by parameters such as the type of material used as the structural matrix (charge distribution, molecular mass, and conformation), film developing conditions (concentration, pH, solvent, temperature, etc.) and category and concentration of additives (antimicrobials, crosslinking agents, plasticizers, antioxidants, etc.) [43].

Until recently, conventional synthetic materials have become part of most materials in our lives, including those present in beverages and food, clothes, daily used instruments, and baby-toys, and even in biomedical applications such as surgical equipment, drug delivery systems, and cosmetic personal care materials. Some studies have associated these materials with potential adverse health problems, particularly in pregnant women and newborn infants. To this end, hormonally active agents are a group of polymeric chemicals that have been associated with critical health issues such as cancerous tumors, congenital disabilities, and other disorders [40]. Furthermore, people have become more aware of the effects of chemically derived compounds and are more cautious in their use of conventional synthetic materials due to their effects on health and the environment. Today’s consumers are more informed and sophisticated in their preferences and choices, increasingly looking for natural and vegan alternate products with high biocompatibility and low environmental implications. Furthermore, increasing efforts and research on the management of plastic waste on Earth are aimed towards finding eco-friendly alternatives to plastics [5]. Such eco-friendly alternatives can be represented by biopolymers, which are disposed in the environment and easily degradable through the enzymatic actions of microorganisms [44].

Compared with conventional synthetic materials that have a simpler and more random structure, these biopolymers are complex molecular assemblies that adopt defined and precise 3D configuration and structures [45]. Based on the composition and chemical structure of biopolymers, they are almost identical to the macromolecules of the native extracellular environment [46]. Many characteristics differentiate between the two types of materials, which are summarized in Table 1. Biopolymers have multiple advantages over conventional plastics due to their low/no toxicity, biodegradability, sustainability, biocompatibility, and extreme hydrophilicity. Furthermore, their morphology and chemical modifications can have a significant impact on their rate of biodegradation [47], an important feature in the development of new applications for food, biomedical, and pharmaceutical industries. Conversely, synthetic materials have a low cost and high thermal and mechanical properties that make them more usable than biopolymers.

Some applications of biopolymers have used mixtures with synthetic materials (such as polyethylene and polyvinyl alcohol), plasticizers (sorbitol and glycerin), nitrogenous bases, and others, thus obtaining a partially biodegradable material [7].

Although biopolymers have many advantages, there are a number of limitations in their processing, starting from the extraction and all the way up to the final product. First, being a completely natural product, biopolymers’ final properties depend largely on the raw material. This can vary greatly due to the origin, climatic conditions, location, harvesting, and processing. Therefore, the world production of biopolymers cannot always maintain the same sustainability. To date, no universal acceptable procedures have been developed for the collection and manufacturing of biopolymer powders from vegetable materials. This is important both for the safety as well as the quality and performance of the final product. Second, because the production of biopolymers is still in its infancy, the production costs are quite high [55]. However, the elimination of recycling and waste taxes through world legislation mitigates some of the high costs. Third, the production of biopolymers necessitates special equipment other than those currently used. The development of such equipment and protocols requires time, additional costs, and trained staff. However, given that biopolymer processing technology is relatively easy and accessible, some existing equipment has been adapted for this purpose, thus reducing the costs [56]. Finally, the lower performance of biopolymers compared to conventional materials may limit their use, although continuous research advances in the technology and material combinations show great improvements in their characteristics that are comparable to conventional materials currently used [57].

## 3. Applications of Biopolymers

**Figure 2:**
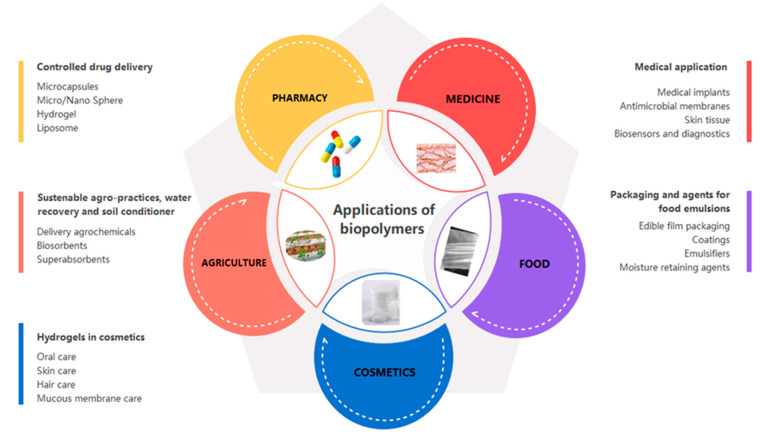
Applications of biopolymers.

Recent research demonstrates the potential applications of biopolymers as materials for manufacturing medical devices [58]. The most suitable characteristics for suggesting these biomaterials are molecular weight, lubricity, material chemistry, water absorption degradation, shape and structure, solubility [59], hydrophilicity/hydrophobicity [60], erosion mechanism [61], and surface energy [62]. Besides these, other applications of biopolymers, such as those presented in Figure 2, are found in industries such as pharmaceutical preparations with encapsulation; food (edible film packaging and emulsifier) [63]; agriculture, which includes sustainable activities, methods for water recovery, and materials used as soil conditioner; the cosmetics industry (especially hydrogels) [64]; and water treatment substances, biosensors, and even data storage elements [1]. In these industries, polysaccharide-based materials have been developed under different forms such as films, membranes, fibers, hydrogels, food casing [65], sponges, and air gels [66].

Packaging in the bio-medical industry is a method that allows for the closure of a pharmaceutical product from its fabrication to its end use. In pharmaceutical packaging, biopolymers are used to protect pills, nutraceuticals, drugs, surgical devices, powders, and liquids [63]. Pharmaceutical packaging has an impact on the isolation and ensures the safety, identity, and convenience of using the products. Packaging should be compatible with the patient’s condition, be free of adverse effects on his/her health, and be environmentally safe [64].

As mentioned above, biopolymers can be also used in the preparation of edible packaging films for food products [65]. These films made from biomaterials can be ingested with the food because they are prepared from polysaccharides and proteins. Edible films have received special attention in the last years because of their alternative potential to replace synthetic materials, which could minimize packaging waste and reduce environmental pollution [66]. As a food packaging material, it can also improve the antimicrobial effect of packaging [67], shelf-life heat resistance, flexibility, mechanical strength, and barrier properties [68]. Edible films/coatings are currently used in a variety of other applications including collagen casings for sausages, chocolate coatings for fruits, and coatings for chocolates and other items [69]. Furthermore, biopolymers are used as emulsifiers and as both thickening and moisture-retaining agents in the food industry with the goal of improving the stability and physicochemical properties of food emulsions [70,71]. Finally, biopolymers have been extensively used in the delivery of bioactive compounds such as probiotics that are susceptible to degradation during preparation, storage, or under the adverse environmental conditions of the human gut. Similarly, they have been used for applications in the pharmaceutical industries as a delivery agent to improve drug stability and bioavailability. In the following sections, we will discuss the applications of biopolymers in drugs and probiotics delivery.

### 3.1. Biopolymers in the Pharmaceutical Industry

**Figure 3:**
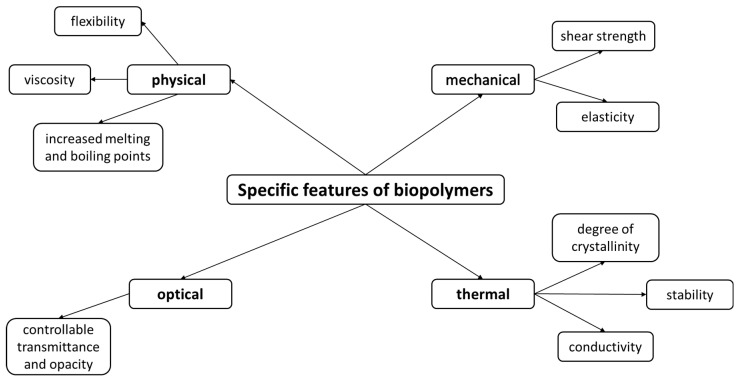
Specific features of biopolymers.

Due to their special properties, biopolymers have slowly begun to replace conventional materials. Whereas in the beginning they were mainly used in the food industry, their application in other related industries took place relatively quickly. In the pharmaceutical industry, they were initially used for the same purpose as in the food industry, which is as thickening and emulsifying agents, host molecules, bulking agents, or fibers. In addition, their use in cosmetics has increased substantially. According to existing data, it is estimated that the world biopolymer market will reach approximately USD 10 billion by the end of 2021, an increase by approximately 17% between 2017 and 2021. The largest market segment is owned by Western Europe with approximately 41.5% of the global market [72]. In biomedicine, polymers have been used successfully both experimentally and in in vivo applications, wound dressing, tissue engineering, drug delivery, or in medical devices such as electronics, sensors, and batteries. Furthermore, due to their physical, thermal, mechanical, and optical properties, biopolymers are ideal materials widely used for food and pharmaceutical applications [5] (Figure 3).

The composition and matrix of biopolymers can be manipulated in order to obtain the appropriate functional properties such as microstructure, permeability, and chargeability that are dependent on the internal structure of the polymer. Electrical characteristics influence the bonding of particles in the biopolymer matrix and their capacity to aggregate. The biopolymer fractions that prevent aggregation are the ones with a high electrical charge [58]. Based on these properties, biopolymers are used successfully to obtain nanoparticles, nanoemulsions, nanogels, or hydrogels with applications in the biomedical industry as carrier systems. Among them, polysaccharides are the most widely used category of biopolymers, either individually or in mixtures with other biopolymers to replace the synthetic materials or exist in addition to them.

#### 3.1.1. Biopolymers for Controlled Drug Release

**Figure 4:**
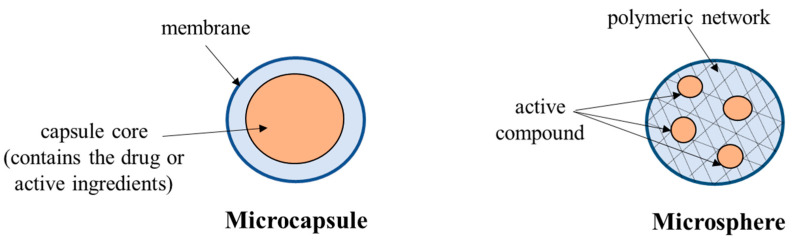
Graphical representation of drug encapsulation (adapted from Madene et al. [74] with permission from the publisher).

Encapsulation involves the protection of living cells from destruction by entrapment in biopolymer membranes and it is applied in micro and macrocapsules [73]. It is the procedure by which one or more materials, representing the active part or core material, is embedded or coated with another material or system, which is actually a mantle, shell, carrier, or encapsulant [74] (Figure 4).

A specific feature of macrocapsules is the relatively large difference between the surface area and the volume. Thus, it is necessary to use a large number of nutrients to obtain an appropriate diffusion gradient for the entry of nutrients. This aspect overlaps with the necessary nutrition for the cells. In macrocapsules, living cells are entrapped in large diffusion chambers formed as flat sheets, hollow fibers, and disks with semi-permeable properties [75]. Macrocapsules can be used as intra or extra-vascular devices [76]. In intravascular devices, cells are connected to the bloodstream as a shunt, oriented outside the artificial capillaries. They are found in the vicinity of blood circulation, assisting with the rapid transfer of therapeutic and nutritional substances such as oxygen. The biggest disadvantage is the potential for developing thrombosis. Therefore, research is moving towards their use as extravascular devices with cells entrapped within semi-permeable diffusion chambers and placed transdermally or in the peritoneal cavity without the need for direct circulatory access. This involves a minor surgery and permits a quick and easy substitution in case of graft failure or when the transplant has to be replaced for other reasons [73]. Microcapsules allow for a fast transfer of beneficial substances and accurately mimics the release of substances such as glucose or insulin. Due to their benefits, most studies focused on developing microcapsules with low or non-inflammatory responses. This feature is used successfully in the treatment of endocrine diseases [77].

Many biocompatible polymers have been used as encapsulation materials. For this, a biopolymer must meet certain criteria: (i) stable and not interacting with the drug it contains; (ii) not interfering with the function and cellular viability; non-toxic, inexpensive, and biodegradable; (iii) both the biopolymer and its degradation products must be non-antagonistic to the host; (iv) molecular weight, solubility characteristics, glass transition temperature, microstructure, and chemical functionality should allow for proper drug diffusion and release; (v) biosafe and biocompatible; and (vi) when biocompatibility needs to be improved, the biopolymer should be combined with other compounds for a synergistic effect.

Depending on the mechanism that controls the release of the active agent from the delivery system, the controlled-release modalities may be different. Thus, biopolymer erosion, diffusion, and swelling, followed by diffusion or degradation, may occur [78]. The erosion mechanisms involve: (i) hydrolysis of hydrogels, an important feature for the controlled release of macromolecules; (ii) solubilization of water-insoluble biopolymers by reactions with groups pendant from the polymer covalently bonded atoms; and (iii) cleavage of hydrolytically labile bonds within the biopolymer covalently bonded atoms. The diffusion process occurs when an encapsulated drug or other active agent crosses the outer membrane of the capsule through the biopolymer used for the controlled-release device. In the case of diffusion-controlled systems, the drug delivery system must be stable in the biological environment and must maintain its size and shape through the swelling or degradation [79]. For example, when biopolymers are combined with other bioactive agents, the drug must be able to diffuse through their molecular structure or through pores when it reaches the biological environment. At this stage, it is very important that there are no changes to the biopolymer itself. Swelling-controlled release devices are those systems that, although dry in the initial phase, will swell when they reach the body and come into contact with fluids or water. The swelling ability of the biopolymers can be triggered by changing the environmental conditions of the delivery system. This is one of the most important and useful features of the biopolymers because, by changing the pH or temperature, the release of drugs or incorporated active substances can be controlled [80]. Finally, the biodegradation of a biopolymer in the body is a natural process through which the active ingredient is completely eliminated.

Synthetic polymers have long been of interest for use as encapsulating agents of various therapeutic substances. Although they show improved pharmacokinetics compared to small molecule drugs, their accumulation in the body has raised toxicity issues [81]. With the reorientation of the medical industry towards the use of biopolymers, the major issue is the selection of the right compounds based on the need and desired effects. Not all biopolymers are suitable as encapsulating agents for drugs. It is important that they release the active substance to the target area at the right time in a safe manner and without side effects, especially considering that the predominant routes are oral or intravenous administration [82]. The most used biopolymers and which are the focus of this review are those based on polysaccharides, such as sodium alginate, chitosan, agar, starch, and cellulose. They react synergistically with other biopolymers and polymers, have low toxicity and non-immunogenic behavior, and are compatible with tissues and cells. These polysaccharides are stable in vitro and in vivo, and are used in the development of microcapsules, microspheres, or nanocapsules. When tested in vivo, they showed high biocompatibility and biodegradability, facilitating treatment, minimizing side effects, and improving the health condition. Their high solubility is a plus for their use as disintegrants in water-soluble tablets. For example, when used in tablets, the coating of chitosan and starch improved their visual appearance, protected the drug from degradation, and masked the unpleasant taste of the incorporated substance [83]. When used as capsule material, gelatin was replaced with alginate, a vegan version, or with cellulose, for hard capsules. The main biopolymers that are widely used and presented in this review are alginate, chitosan, agar, starch, and cellulose.

##### Alginate and Its Use for Drug Delivery

Alginate is probably the most widely used biopolymer in the pharmaceutical and medical industries. This is due to its special encapsulation properties and role in wound healing. It was first isolated in the 1980s and since then it became a multifunctional compound in many applications. Thus, it is obtained at low cost and is a renewable and readily available, biodegradable, non-toxic, biocompatible, and, importantly, mucoadhesive and non-immunogenic compound [84]. It is recognized in the pharmaceutical industry as an excipient and is used to treat reflux esophagitis [85].

Structurally, alginate is a hidrosoluble polysaccharide formed from alternative blocks of 1–4 linked α-l-guluronic acid and β-d-mannuronic acid residues. It contains varying lengths of G-blocks, M-blocks, and/or MG/GM-blocks. High G content alginates have the ability to form stiffer, brittle, and more porous gels, but with increased strength, while high M content alginates tend to obtain more elastic and weaker gels [86].

Alginate is obtained from brown algae and is found as alginic acid sodium, calcium, and magnesium salts. The algae species used for the extraction of trading alginates are *Macrocystis pyrifera, Laminaria hyperborea, Saccharina japonica*, and *Ascophyllum nodosum*. It can be synthesized by various species of bacteria such as *Azotobacter vinelandii* and various *Pseudomonas* species, although they are not commercially available [72]. Alginate extraction is achieved by a relatively simple process. First, the raw material from the algae is ground and washed with acid followed by the extraction with hot alkali. The alginic acid is obtained after the extract has been filtered, precipitated with calcium, and acidified. The required salt form of alginate is obtained by treating insoluble alginic acid with metallic carbonates, oxides, or hydroxides [87]. Alginate biocompatibility has been extensively studied, with data showing that oral administration of alginate does not trigger many immune responses, in addition to the finding that it is non-toxic and biodegradable [88]. By contrast, intravenous administration of most commercial alginates can lead to adverse body reactions and fibrosis [89].

As an encapsulating agent, alginate was first used in the treatment of diabetes in the encapsulation of pancreatic islet cells [90]. Since then, it has been used for both macro and microencapsulation, and for other endocrine and recombinant cells for the release of therapeutic gene products such as growth hormones or human clotting factor IX (Table 2). It is also used in bioartificial organs such as they kidneys or for the protection of hepatocytes or parathyroids. Alginate gels cannot provide immunoprotection because they are too porous. Therefore, in most applications, alginate gels must be coated with cationic polymers of synthetic origin. For alginate-based coatings, the most used cationic polymers are poly-L-lysine and poly-L-ornithine, but lately polyethylene glycol (PEG), glutaraldehyde, chitosan, and agarose have also been applied. Occasionally, other substances are used to reduce permeability, to ensure mechanical stability, and to increase the durability of the capsules; however, PEG remains the most used coating material. Another way to stabilize alginate gels is through the application of covalent crosslinking molecules, although this method of encapsulation interferes with the functional viability of the cells and can lead to cell toxicity [91].

Thus, based on its characteristics, alginate seems to be the most suitable biopolymer used for drug encapsulation (Table 2). This is due to its specific properties, especially as a matrix for controlled drug delivery devices. In addition, alginate is cheap and readily available, is accepted for consumption in quantum statis doses, is nontoxic, and ensures the protection of the mucous membranes of the upper gastrointestinal tract [101].

##### Chitosan and Its Use for Drug Delivery

Chitosan is a polysaccharide found in shellfish, fungi, annelids, mollusks, and insects. It is the second most outspread natural polysaccharide on Earth, after cellulose. Commercially, it is produced from chitin, being a poly β (1 → 4) -2-amino-2-deoxy-β-d-glucan deacetylated chitin. It has a strong affinity for polyanions, contains reactive NH_3_^+^ and OH^−^ groups, and is soluble in acidic aqueous solutions. It is nontoxic, odorless, bio-compatible, and biodegradable. Due to its antibacterial properties, chitosan is used for microencapsulation, in particular for cells that require a cationic environment. Numerous applications in drug delivery include drug targeting systems for oral, nasal, ocular, and transdermal routes [102]. For this purpose, chitosan has been used in the development of gels, films, oral tablets, beads, and microspheres [103].

The capacity of chitosan as an encapsulating agent is greatly influenced by its molecular weight, degree of deacetylation and crystallinity, and extent of ionization/the free amino group. Thus, when the amino group at the 2-position of glucosamine units of chitosan is the main site for the immobilization of thiol groups, it results in thiolated chitosan. The thiolated chitosan derivatives are chitosan-cysteine, chitosan-thiolactic acid, chitosan-thioglycolicacid, chitosan-homocystenine, chitosan-N-acetylcysteine, and chitosan-glutathione. The thiolated chitosan has been used for anticancer drugs because it offers efficient mucoadhesivity, membrane permeation, and an enhancing capability and improved inhibition for P-glycoprotein [104]. Phosphorylated chitosan and its derivatives have different features such as high hidrosolubility and a metal chelating tendency, used in tissue regeneration, drug delivery intermediates, fuel cells, and in the food industry [105].

Structurally, chitosan is composed of free amine groups in media with a pH over 7.5 and protonated amines are formed in media with a lower pH. These pH-sensitive characteristics make chitosan-based compounds suitable in controlled-release technologies. Under well-established conditions, chitosan microcapsules containing the drug as an active ingredient permits its slow release at the target site [106]. For example, when encapsulated in chitosan, lipophilic drugs were effectively released into the intestinal tract [107]. When used as a vehicle to encapsulate vaccines, it allowed for their controlled release and delivery to targeted sites [108].

There are a number of advantages in using chitosan in the pharmaceutical industry for drug delivery, such as: (i) the controlled release of encapsulated substances; (ii) the elimination of toxic agents in the development process (due to dissolution in aqueous solution); (iii) crosslinking readily available free amino groups; and (iv) improved membrane absorption by mixing cationic chitosan with an anionic material [109]. When used as a coating agent for nanoparticles for the treatment of brain disease, chitosan protects against enzyme degradation, controls release, and improves bioavailability. In addition, it enhances drug permeability across the blood–brain barrier by affecting tight junctions [110]. Equally important are its hemostatic, bacteriostatic, anticholesterol, anticarcinogenic, and fungal characteristics.

In addition, chitosan has good bioadhesive properties and slows down the drug release in the nasal cavity, thus increasing bioavailability and the transfer of drugs from the nasal cavity to the brain [111]. When chitosan was used in membrane development, it increased permeability to acidic drugs. It is insoluble at a pH greater than 6.5 and prevents the burst effect of the release in the first segments of the gastrointestinal tract [112]. It has also been used successfully for antiviral and antibiotic encapsulation, as seen from Table 3.

Chitosan has been extensively used as a matrix for extended drug release, especially due to the simple obtaining procedure, low cost, and biocompatibility. The biocompatibility of chitosan also derives from the fact that it is already part of the human food chain due to its presence in numerous fungi [123]. Chitosan increases the solubility of insoluble drugs when used in mixtures with inorganic nanoparticles, forming a stable complex with safe delivery to the specific site. It was effective when encapsulated hemoglobin, astaxanthin, quercetin, vaccines, or vitamins. Besides its applicability in drug delivery, chitosan is also used in wound dressing, tissue engineering, bioimaging, biosensors, and packaging, among other uses [124].

##### Agar and Its Use for Drug Delivery

Agar is a long-chain biopolymer obtained from species of algae from the Rhodophyceae class, most commonly found in *Gelidium sp.* and *Gracilaria sp.* It represents the supporting structure of algae and is composed of a mixture of agarose and agaropectin, the gelling and the non-gelling fraction, respectively [125]. Agaropectin is usually removed during processing in order to obtain an agar powder with higher gel strength. Agarose is composed of repetitive units of d-galactose and 3-6, anhydro-l-galactose, linked by alternating α- (1 → 3) and β- (1 → 4) glycosidic bonds.

The ratio of agarose to agaropectin depends largely on seaweed growth, the environmental condition of seaweed growth, extraction methods, and rheological and gelling properties. These changes affect the final mechanical properties of the gels [126]. Agar quality can be significantly improved by modification, which is the most widely used chemical method. It involves hydroxypropylation, acetylation, etherification, and oxidation, the last one being the most commonly used [127]. Due to its gelling capacity, gel reversibility, and high hysteresis, agar is intensely used in various applications, mainly in the food industry, due to its ability to form gel and have an odorless taste. The most important agar evaluation index is gel strength, an important feature for pricing and developing new applications. The easiest way to improve agar characteristics is to remove the sulfate groups with hydrogen peroxide. Thus, after modification, the viscosity, ash content, and sulfate content decreased. Conversely, the gel strength, whiteness, and transparency increased after modification, in contrast to gelling, melting, and dissolving temperatures that decreased after modification [128]. Unlike other biopolymers, agar has been widely used as an encapsulating agent for probiotics since 1988 [129]. The method followed a simple way of encapsulation, which involves the use of drug microparticles and their dispersing at high temperatures in a hydrophilic liquid vehicle. After cooling, due to the transition to ambient temperature, the beads solidify. The same encapsulation method is currently used both for agar and other biopolymers used for this purpose [130]. The method depends on dropping a hot hydrophilic polymeric solution on the top of a cooled organic liquid, such as ethyl acetate which is a non-toxic compound, during which the polymer and the incorporated drug are insoluble. Usually, when only agar is used as an encapsulating agent, the release of the drug occurs in two phases. The first and faster phase leads to the release of 10–20% of the drug, based on the agar content of the beads. The second is a slower and more prolonged phase and becomes even slower as dissolution proceeds. In the first phase, the drug presents in a molecular state on the surface and is released in the outer layer of the bead so that in the second phase, its release is due to dissolution from the solid core. When used as an encapsulating agent, the larger the mass of agar in the beads, the denser the matrix formed and the lower the transfer of drug molecules through the beads. Similarly, the beads that contain a lower percentage of agar in the composition have a higher water content, which explains the rapid rate of drug release [131]. Therefore, agar can be used for the development of sustained-release dosage systems because it is a natural, inert, non-toxic, renewable, biocompatible, and inexpensive material.

##### Starch and Its Use for Drug Delivery

Starch is one of the most abundant renewable biopolymers on Earth and is non-allergenic, GRAS (generally recognized as safe), and cheap [132]. It is found in peas, corn, rice, wheat, potato, and beans [133]. Starch granules vary in size, shape, particle size distribution, and in the amylose–amylopectin ratio depending on the botanical origin and maturity [134]. The high encapsulation efficiency was reached when the amylose:amylopectin ratio was 25:75 [135]. Starch granules are composed of amylose and amylopectin, free fatty acids and lysophopholipids, proteins, phosphate esters, and water [136]. Amylose is the linear fraction and is composed of glucopyranose units linked by α-(1,4)-glycosidic linkages, while amylopectin is a highly branched polymer with short α-(1,4)-glycosidic chains linked by α-(1,6)-glycosidic branching points [137]. Although amylopectin has a high viscosity and is a good thickening agent, it produces very weak gels with poor mechanical properties [138]. Starch is a biopolymer available in the form of powders, hydrogels, films, and sponges [139]. Due to its low cost, physicochemical features, biodegradability, and biocompatibility, native and modified starch has been widely used in the food, chemical, pharmaceutical, and environmental industries [140].

In the pharmaceutical and medical industries, starch has been used as a pharmaceutical excipient, a tablet super disintegrant (immediate release tablet formulations), and a controlled/sustained-release polymer or as plasma volume expander, useful for patients suffering from trauma, heavy blood loss, or in cancer treatment [141]. Research has focused on the ability of native starch to be dissolved by pancreatic enzymes after oral ingestion, followed by absorption from the small intestine into the systemic circulation. There is also a resistant part of starch that is not digested in the small intestine and is fermented by colonic bacteria. When used as an encapsulant for drugs, it is combined with other biopolymers precisely to limit or attenuate enzymatic degradation in the stomach, thus facilitating the absorption of an adequate amount of the therapeutic agent [142]. In pharmacotherapy, the main objective of such a system is to provide controlled drug release and prevent fluctuations of active substances in the blood in order to maintain drug plasma concentration within the optimal range, in accordance with therapeutic recommendations.

Starch with high crystallinity levels has been explored as an encapsulation matrix. In order to be used successfully in drug delivery and other industries, starch can be modified so that the physicomechanical properties are adjusted to maximize its use. Starch can be modified by chemical, physical, enzymatic, and genetic processes. Of these, chemical process is used most frequently due to its non-disintegrating nature and potential increase in the functionality of the modified starch. The applications of starch as an encapsulating agent of active substances are presented in Table 4.

Most starch-based drug delivery systems have been developed with starches extracted from potato, maize, corn, cassava, and wheat [118]. As shown in Table 4, starch is a viable source of biopolymer, used as an encapsulating agent for controlled drug delivery systems. In its unmodified form, starch is not as effective as a drug delivery system due to poor mechanical properties, such as low shear stress resistance or high retrogradation and syneresis, thermal decomposition, reduced processability, and solubility in common organic solvents [156]. However, after modification, starch can be used successfully for this purpose. For example, modifying starch in order to obtain resistant starch has led to its use for improving the gut microbiota population with a role in modulating signaling pathways associated with anti-inflammation, anti-diabetes, and anti-obesity [157]. Resistant starch, due to its high amylose content and low amylopectin, has been recognized as a healthy food for humans and animals. It can be considered prebiotic and may reach the colon due to its resistance to digestion by pancreatic enzymes in the small intestine [158]. Therefore, encapsulation has been suggested as the best approach to improve prebiotic–probiotic symbiosis.

##### Cellulose and Its Use for Drug Delivery

Cellulose, a natural polymer, is the most renewable and abundant polysaccharide. Cellulose has been used as an immunoprotective macrocapsule because it does not form a hydrogel and it is mostly applied in inert diffusion chambers. As an encapsulating agent, it is beneficial for cytotoxic epithelial cells in the treatment of pancreatic cancer, insulin-producing cell lines (HIT-T15), embryonic kidney cells, and hybridoma cells. It is recognized as a new nanovehicle for oral colorectal cancer treatment with high drug release at a neutral pH compared to acid pH, being proposed as a safe oral delivery system for controlled colon cancer treatment [159].

Cellulose is the structural part of the cell wall of green plants, algae, or oomycetes. It is part of the polysaccharide group and is composed of a linear chain of β (1 → 4)-linked d-glucose units. Considering it has an amphiphilic character, it can be used as a surfactant and/or stabilizer at the water–oil interface in pickering emulsions [160]. Cellulose is insoluble in water and most organic solvents. The cellulose derivative, carboxy-methyl-cellulose (CMC), contains carboxymethyl groups bound to the OH-groups of glucopyranose monomers on the cellulose backbone. CMC is mostly applied as a matrix molecule and, in order to ensure mechanical stability and immunoprotection, requires surface coating. CMC has been used as an encapsulating agent for probiotics, but due to the hydrophilicity of the cellulose derivatives, physical degradation occurs when passing through the digestive system. Combined with alginate, it provides a better medium system for probiotics with enhanced tolerance at low pH and a more durable delivery of probiotic cells. Long-term storage stability depends on low water activity and low temperature. The most used dehydration methods to reduce water activity are freeze-drying, spray-drying, vacuum-drying, convective air-drying, and fluidized bed-drying. Among all, freeze-drying is the best method for preserving cells’ viability because it reduces the damage to biological structures by eliminating water through sublimation [155].

Cellulose crystals have been used in combination with chitosan to encapsulate vitamin C. Stability of vitamin C is highly dependent on light, pH, and the dissolved oxygen in the environment, but is maintained due to the encapsulation with cellulose and chitosan crystals, and this may be a way to preserve highly unstable compounds during long-term storage in functional systems [161]. Similarly, nanofibrillated cellulose, combined with soybean oil-in-water emulsion and whey protein isolate, was used to encapsulate vitamin D3. Vitamin D3 encapsulation efficiency has improved with increasing emulsifier concentrations. Increasing the concentration of nanofibrillated cellulose has improved the stability and efficiency of encapsulation against environmental stresses (pH changes, salt addition, and thermal processing). The procedure may be the basis for more suitable encapsulation technologies for liposoluble vitamins in emulsion-based food products [162].

In addition to encapsulating vitamins, cellulose and cellulose derivatives have been used as agents to encapsulate drugs and probiotics with active substances. For example, ethyl cellulose nanoparticles have been shown to be effective in encapsulating clarithromycin (3:1 weight ratio of ethyl cellulose:clarithromycin). Once encapsulated, clarithromycin was more effective against *Helicobacter pylori* gastric infections. Tests performed in vivo on laboratory mice have clearly indicated better elimination of bacteria from the stomach by encapsulated clarithromycin compared to the nonencapsulated drug [163]. Ethyl cellulose and microcrystalline cellulose were also used for the encapsulation of antihypertensive drugs. This is important considering that, in the standard method of manufacturing microspheres involving emulsification and solvent evaporation, the solvents used are usually dichloromethane or chloroform, which are hazardous for the environment. Therefore, less toxic substances such as ethyl acetate are used to prepare the microspheres. Furthermore, the drug release from the microspheres is faster than the tableted ones, suggesting that tableting of the microparticulate systems may be optimal [163].

### 3.2. Biopolymers in Probiotic Encapsulation and Delivery

Due to their special properties, biopolymers have been used to encapsulate probiotics. Probiotics are living organisms with benefits on the hosts’ health if ingested in adequate amounts [164]. According to the International Scientific Association for Probiotics and Prebiotics (ISAPP), a sufficient amount of probiotics with a beneficial effect on the hosts’ health involves ingesting 1 × 10^9^ CFU per serving [165]. Unlike probiotics, prebiotics are nutrients, usually high-fiber foods, providing the substrate that is selectively utilized by the hosts’ microorganisms, conferring a health benefit [165]. Most probiotics in the human body form the commensal intestinal microbiota with a role in increasing resistance to infections and boosting host immune system, glucose and lipid metabolism, degradation of complex carbohydrates, and synthesis of vitamins and bile acid [166]. Although the effects of probiotics on various diseases is still debatable, several studies showed beneficial effects in the treatment and prevention of infectious diseases. For example, strains of *Lactobacillus plantarum*, *Lactobacillus casei*, or *Lactobacillus paracasei* had antifungal, antibacterial, and antioxidant effects. Other strains have been shown to have anti-inflammatory effects, to lessen the risk of osteoporosis, maintain cholesterol levels, and prevent the proliferation of cancer cells [167]. Therefore, due to their beneficial effects and health claims, there has been a worldwide explosion of probiotic-based health products in the form of dietary supplements [168]. As such, the global probiotic market is soon reaching USD 50 billion and, with that, the range of probiotics-containing products and associated health claims continue to expand rapidly. Currently, in Europe, the probiotic market is subject to regulatory requirements and compliance with rules and regulations in order to meet certain standards for product registration and use [169].

The use of encapsulation technologies of probiotics has been intensely studied in order to increase probiotics’ viability throughout manipulation, storage, commercialization, and incorporation in food and pharmaceutical products so that these cells are viable during their transit and residence in the gastrointestinal tract. Therefore, improving probiotic survival and resistance to adverse conditions through encapsulation is paramount to their effectiveness in health and disease conditions. To prove their effectiveness, encapsulated probiotic strains have been incorporated into a wide range of food products such as yoghurt [170], cheeses [171], frozen dairy desserts [172], beverages [173], and meat products [174], increasing their therapeutic effects [168]. Once encapsulated, probiotics embedded in food matrices maintained their viability even two months under refrigeration [175]. They can also be mixed in a single microcapsule or in dual core capsules with separation microcompartments [176] and a combination of at least two strains can improve their effect [174]. In order to scale up production, several industrial partnerships between food producers and probiotic companies have been formed. For example, Christian Hansen and Dos Pinos developed probiotic ice cream, Balchem Encapsulates and Rosell Institute developed probiotic raisins and bars, and Dannon uses probiotics encapsulated in their products [177]. Encapsulated probiotics have been effective in irritable bowel syndrome [178], colitis, abdominal pain [179], and other gut or metabolic conditions characterized by microbiota dysbiosis [180].

Several encapsulation methods have been developed and used.

(1) Microencapsulation represents a physicochemical or mechanical process used to trap a substance (active agent) into a coating material (defined as wall material). In this way, spherical particles have a thin and strong but semipermeable membrane with a diameter from nanometers to a few millimeters [174]. The purpose of the procedure is to protect compounds or viable cells against environmental agents that can destroy the core [181].
(2) Spray-drying technique is suitable for industrial applications on a large scale, involving atomization of a liquid mixture and the solvent is evaporated at contact with hot air or gas.
(3) Lyophilization involves freezing the cells with the material used for encapsulation (usually at freezing temperatures), followed by vacuum elimination of water at a pressure between 0.05 to 0.1 mBar and temperature between −50 °C to −30 °C. To preserve and stabilize the activity of lyophilized probiotics, cryoprotectants are added, such as lactose, trehalose, sorbitol, sucrose, milk protein, or skim milk.
(4) Extrusion is the most common technique to use biopolymers as encapsulation materials. The method involves obtaining a hydrocolloid solution, followed by the addition of microorganisms, formation of droplets using a syringe needle (pilot scale) or an extruder (industrial scale), and their release into a hardening solution (typically calcium chloride) [182].
(5) Emulsion is when a small volume of a hydrocolloid suspension containing microorganisms (discontinuous phase) is added to a larger volume of vegetable oil (continuous phase). Using an emulsifier, the mixture is homogenized. After emulsion formation, it can be insolubilized to manufacture gel capsules. The big disadvantage of this method is that the particles obtained vary greatly in shape and size, although bead sizes can be reduced by mechanical homogenization [183].
(6) Spray–freeze-drying is a combined procedure that involves steps used in lyophilization (freeze-drying) and spray-drying. The advantage is that it provides capsules with a controlled size and higher specific surface area, unlike those obtained by spray-drying. The disadvantages of the method refers to high costs (approximately 50 times higher than the classic spray-drying version), long processing times, and the high-energy requirement.
(7) Layer-by-layer is technology based on alternating coating layers of cationic (e.g., chitosan) with anionic (e.g., alginate) biopolymers on cells via electrostatic interaction [184]. It has the advantage of enhanced bacterial viability throughout the gastrointestinal tract, along with the survival of probiotic cells against acidic and bile salt insults, mucoadhesion and growth on intestinal tissues, and in vivo survival [179].

In addition to ensuring cell viability along the gastrointestinal tract, the stability of probiotics during storage is also very important. In this regard, encapsulation has proved to be an effective method. For this, the material used to encapsulate microorganisms is the first and most important factor in maintaining their viability [185]. It improves the survival of probiotics during manufacturing processes, especially heat processing [186] and storage [187]. An important aspect of this process is cytotoxicity. According to ISO 10993-5 [188], a material used for encapsulation is potentially cytotoxic when cell viability decreases below 70% after exposure [189]. In this regard, polysaccharides such as alginate, starch, chitosan, and cellulose, as well as other biopolymers or chemicals, have low or no toxicity and do not affect cell viability. On the contrary, they maintained cellular stability for a long time, particularly when kept in refrigerated or frozen conditions.

Although the encapsulation method has many advantages, there are still several aspects that must be consider. These are: (i) biosafety concerns preventing clinical translation of the cell microencapsulation; (ii) concerns regarding the manipulation and extraction procedures that must be refined in order to be as minimally invasive as possible; (iii) concerns regarding the optimization of cost effectiveness; and (iv) concerns regarding the consideration of internationally accepted regulations for the use of probiotics. Therefore, applications of biopolymers for the coating of encapsulated strains for the purpose of protection in the intestinal gastrointestinal tract or as carriers for direct encapsulation of microorganisms should involve procedures that facilitate high bacterial viability.

#### 3.2.1. Alginate’s Use for Probiotic Delivery

Among biomaterials used for encapsulation, alginate is the most widely used due to its strong gelling properties and ability to coat within a short time. Additionally, as a dietary fiber, alginate strengthens the functionality of probiotics used in several diseases such as diabetes or obesity [190]. As seen from Table 5, alginate has proven to be a good microencapsulation agent by extending cell viability in refrigeration and freezing conditions, as well as in adverse gastric and intestinal environments.

Alginate is hemocompatible, does not accrue in organs, is water soluble, biodegradable, and can form gels under mild conditions. It develops gel at ambiental temperatures and prevents the destruction of the activity of thermolabile drugs. By cross-linking with other agents, it forms insoluble gel that delays the drug release. However, alginates have low mechanical properties, therefore they must be reinforced by combining with other biopolymers or with various conjugates in order to obtain both ionically and covalently cross-linkable capsules.

#### 3.2.2. Chitosan Use for Probiotic Delivery

Like other polymers, chitosan has been used to encapsulate probiotics. The best-performing formulas were identified in combinations of chitosan with other biopolymers such as alginate, agar, or gelatin. The most used combination is with alginate, in which chitosan is used as a final layer of microcapsules (Table 6). This is because at pH 7, chitosan that is positively charged develops strong bonds with gelatin and agar, which are negatively charged [203]. What sets chitosan apart is its antibacterial properties as it is a cationic polysaccharide. The disadvantage of chitosan, however, is the need for solubilization in an acidic environment. Usually, acetic acid is used to solubilize the powder and obtain the coating-forming solution without turbidity, which occurs when a compound has not been fully solubilized [204]. Chitosan cannot be used individually as an encapsulating agent with a role in maintaining cell viability. This is because it increases cell membrane permeability, leading, in the end, to cell loss [205]. Due to this, chitosan is mixed with other natural substances when used as an encapsulating agent.

Although the effectiveness of chitosan encapsulation has been demonstrated, it does not appear to be the best biopolymer for probiotic encapsulation. Besides the fact that it cannot be used individually and produces turbidity, the obtained microparticles are usually larger in size, more porous, wrinkled [213], sticky, and the aggregation is worsened [211]. However, utilization of chitosan should not be limited as chitosan improves potential bioadhesion and facilitates the controlled release of bacteria [213].

#### 3.2.3. Agar Use for Probiotic Delivery

Agar is one of the polysaccharides intensively used to obtain tablets or other formulas of drugs released in the gastrointestinal tract, but is less used as an encapsulating agent for probiotics. Although there have been attempts to microencapsulate with agar, when used in combination with other biopolymers (Table 6), research on its use is limited. This is due to the higher ability to obtain films and lesser ability to facilitate the development of coatings. These applications are mainly due to the ability of agar to form viscous solutions by solubilizing the powder in water at very high temperatures (over 90 °C) in order to obtain a termoreversible gel. Agar cannot produce gel at lower temperatures and high temperatures affect the viability of microorganisms. To date, no working method that could involve the solubilization of agar powder in liquids with lower temperatures has been developed. When used as an encapsulating material for essential oils, the temperature of the film-forming solution is lowered to 40 °C, after which essential oils are incorporated [216,217]. Agar-based films have low mechanical properties such as low tensile strength and poor elasticity. Therefore, in the development of films, it is preferred to mix it with other polysaccharides, proteins, or lipids.

#### 3.2.4. Starch Use for Probiotic Delivery

Starch can be used as an encapsulating agent but it has weaker characteristics than alginate, chitosan, or cellulose. In the pharmaceutical field, starch is used mainly for encapsulating drugs or active substances when molded into tablets or oral formulations. This is because starch is strongly hydrophilic and is easily dissolved in liquids at ambient temperatures. However, it has the ability to form very small microspheres constituted in resistant aggregates, allowing for a better protection of the core [218]. For example, when used as an encapsulating agent for *L. plantarum*, rice starch maintained cell viability both at 4 °C (refrigeration conditions) and at temperatures above 50 °C. However, as an encapsulating agent, starch proved to be more effective when mixed with other compounds. For example, the combination of starch and alginate resulted in microcapsules with increased probiotics resistance to simulated gastric conditions (Table 7).

#### 3.2.5. Cellulose Use for Probiotic Delivery

Carboxymethyl cellulose (CMC) is one of the most widely used forms of cellulose due to the fact that it is the most affordable in terms of spread and cost. Although it is a compound that prevents lipid oxidation and reduces oxygen permeability due to the small number of hydroxyl groups in the structure, CMC is a highly water-soluble compound. This limits its use as an encapsulation material for probiotics as it is degraded in the digestive system. Therefore, when used in combination with other biopolymers such as polysaccharides (carrageenan, alginate, chitosan, and starch), proteins (gelatin), or other natural compounds (inulin), it increased the viability of encapsulated anaerobic probiotics by 36% [227]. The microencapsulation characteristics of CMC have been improved due to the addition of other substances such as gelatin or carrageenan. In general, the encapsulated probiotics maintained their viability for 120 min in simulated gastric conditions, regardless of the type of probiotic encapsulated. When lyophilization was used as the encapsulation method, cellulose and alginate maintained the viability of *L. plantarum* for approximately 160 days in refrigeration conditions (Table 8).

## 4. Challenges and Limitations

The rapid adoption of the use of biopolymers is still hindered by several factors. First, research thus far has been conducted primarily in vitro; therefore, more in vivo and clinical trials are needed to demonstrate the health benefits of biopolymers and the biocompatibility in various biomedical applications, in particular when used as encapsulation materials for drug delivery. When used for the treatment of various diseases, more studies are needed to assess the appropriate compound: either alone or in combination to achieve the desired payload in a highly regulated and site-specific manner at therapeutically relevant concentrations [227].

A second challenge is to obtain materials with properties similar or better to synthetic products by improving end-use mechanical properties, kinetics and release, thermal resistance, and barrier properties. For example, some products exhibit low mechanical properties, rapid degradation, and high hydrophilic capacity especially in humid or adverse environments, rendering their application unviable.

Although numerous studies have examined the use of encapsulated probiotics, there is a need for in depth interdisciplinary research that includes microbiologists, medical doctors, and biomaterial, food, agro and chemical engineers. This will lead to better and more efficient prototypes of probiotic encapsulating formulations, to the identification of the most specific/effective probiotic strains, and to the most suitable polymeric carriers applied for product manufacturing. In addition, this will lead to the optimization of the entire process based on the natural characteristics and sensitivity of the selected strain, and will identify ways on how to develop the best formula based on in vitro, in vivo, and pre-clinical techniques considering the release, manufacturing, packaging, transportation, and storage of capsules. Third, the challenges related to costs, economic aspects, and the gap between policy and implementation of the new technologies on a global level need to be addressed in this rapidly emerging field.

## 5. Conclusions

Although research in this area has revolutionized the biomedical and pharmaceutical industries, significant work still lies ahead if we are to effect changes not only at the individual level but also to bring sustained and affordable environmental changes. Biopolymers have proven effective as encapsulation materials for controlled drug release systems. Significant progress has been made on the biocompatibility, biodegradability, and mechanical and thermal properties of the materials involved. However, challenges still remain in developing target-specific carriers that are biocompatible with various delivery routes for providing sustainable release at the target site. Strategies have been proposed to improve stability of polymers, product kinetics, and release time as well as clinical efficiency. For biomedical applications, it is important to develop uniform guidelines for polymer applications in order to improve versatility and safety and avoid contamination. From the evidence presented thus far, it is obvious that among biopolymers, polysaccharides-based applications are the most used in the field due to their protective, physicochemical, and low immunogenicity characteristics. Although they present some limitations, the ability to react synergistically with other biopolymers or other natural or synthetic substances make their applications widely used. More studies evaluating the technical parameter optimization, efficiency of encapsulation with different formulations, and product-loading capacity concerning viability and metabolic activity should be undertaken. For example, studies examining the functional interactions between the polymer networks and the coating materials, in order to improve capsule stability, product metabolic activity, release time, and viability, should be high on the list. Notwithstanding current limitations from the host perspective, the use of polymers, particularly polysaccharides-based, will continue to expand with an eye towards improving polysaccharide-drug interactions, the optimization of pharmacokinetics and pharmacodynamics, and the compatibility of the polysaccharide with the target tissue. Nevertheless, while more research is needed, polymer-based applications are of great benefit for delivering small molecules that are highly effective, biopotent, and safe.

## Figures and Tables

**Table 1:** Characteristics of biopolymers vs. synthetic polymers.

Characteristic of Materials	Biopolymers	Synthetic Polymers	References
Main source	Agro-resources	Petroleum and gas	[48]
Biodegradability/environmentally friendly	YES	NO/slow	[49,50]
Structure	Well defined	Stochastic	[48]
Chemical backbone structure	Carbon, oxygen, and nitrogen	Mostly carbon	[48]
Dispersity	Unity	˃1	[51]
Physicochemical resistance	Low	High	[52]
Toxicity	Low	High	[41]
Thermal stability	Low	High	[52]
Mechanical properties	Low	High	[53]
Sustainability	High	Low	[52]
Availability	High	Decreasing	[52]
Cost	High (depends on the type)	Low	[54]

**Table 2:** Alginate use for drug delivery.

Biopolymers	Entrapped Substances	Applications	Benefits	References
Alginate	Rifampicin	Drug delivery carriers	Nanoparticles are pH sensitive with the highest release of the active substance occurring at a pH of 7.4. Toxicity and safety tests were excellent with no systemic toxicity after oral administration of nanoparticles.	[92]
Alginate	Ibuprofen	Drug delivery system	Controlled drug release was maintained for 4 h (67.53% of the drug formulation).	[93]
Alginate and methylcellulose	Indomethacin	Drug delivery carrier	Controlled drug release. There was no interaction between the loaded drug and the polymers.	[94]
Alginate	Metformin hydrochloride	Drug delivery system	Good release time; microspheres may be used in the treatment of diabetes.	[95]
Alginate	Diclofenac sodium	Controlled-release microparticles	The drug: polymer (ratio 1:3) was obtained by emulsification and the drug release followed zero order kinetics, optimum for controlled drug release delivery.	[96]
Alginate and sodium carboxymethylcellulose	Ceftriaxone sodium	Multiarticulate beads	The use of the biopolymer matrix decreased drug release in gastric conditions but sustained it at intestinal pH. The beads swelled at pH 1.2 but particle diffusion and erosion occurred at pH 6.8.	[97]
Alginate	Furosemide	Controlled drug delivery beads	Drug release was controlled due to the thicker membrane and reduced beads swelling. Release of Furosemide depends on the conditions of the coating treatment.	[98]
Alginate	Isoniazid	Oral drug delivery	Microspheres were present in the intestinal lumen 4 h after administration and were detectable in the intestine after 24 h of oral administration. Approximately 26% of the drug was released in the gastrointestinal fluid (pH 1.2) in 6 h and 71.25% in the simulated intestinal fluid (pH 7.4) in 30 h.	[99]
Alginate	Nicotinic acid	Aerogels	The release of the drug was prolonged when the core was surrounded by several alginate-based membranes. Due to coating, 50% of the drug was released within 4 h.	[100]
Alginate, carboxymethylcellulose, and chitosan	Amoxicillin	Coated beads	In gastric pH conditions, the drug release was prolonged from 61 min to up to 8 h.	

**Table 3:** Chitosan use for drug delivery.

Biopolymers	Entrapped Substances	Applications	Benefits	References
Chitosan	Acyclovir	Drug delivery system	The grafting efficiency was 94% and the slow release of the drug was prolonged to 12 h.	[113]
Chitosan	Chlorhexidine diacetate	Buccal tablets	The tablets dissolved more quickly in vitro than chlorhexidine powder and both maintained and even improved the antimicrobial activity of the drug, particularly against *Candida albicans*, due to antimicrobial activity of the polymer itself.	[114]
Chitosan aspartate, glutamate, and hydrochloride	Vancomycin	Peptidic model drug	The sustained release from the microspheres minimized its solubilization in the upper gastrointestinal tract.	[115]
Chitosan	Tetracycline	Controlled drug system	The concentration of drug released was above the minimum limit required for the inhibition of *Staphylococcus aureus*.	[116]
Chitosan and oleic acid	Camptothecin	Controlled drug system	The encapsulation efficiency was about 78%. When its effectiveness in the simulated gastrointestinal fluids was tested, the drug was released slowly into the gastric environment. In intestinal fluids, the drug release was controlled. The drug embedded in chitosan was 75% protected from hydrolysis.	[117]
Chitosan	Satranidazole	Subgingival films for periodontitis	The drug was released for 96 h; the population of gram-positive bacteria was reduced.	[118]
Chitosan and alginate	Amygdalin	Drug delivery system	The controlled amygdalin release was performed for 10 h; the maximum amygdalin released was 70.46% at pH 3.1, 81.86% at pH 5.0, and 86.03% at pH 7.4.	[119]
Chitosan and graphene	Isosfamide	Sustained drug microspheres	The drug diffusion was the most controlled for when isosfamide was entrapped in microspheres.	[120]
Chitosan and xanthan gum	Ciprofloxacin	Controlled-release hydrogel	The entrapment efficiency of the prepared hydrogel increased with the drug increasing concentration and the maximum was reached at 93.8%.	[121]
Chitosan	Interferon-α	Nanoparticles for oral delivery	In mice, the nanoparticles were found in plasma at 1 h after administration, unlike the commercial interferon which could not be detected.	[122]

**Table 4:** Applications of starch for drug delivery.

Biopolymers	Entrapped Substances	Applications	Benefits	References
Corn starch	Chlorhexidine gluconate	Long-term drug delivery system	In vitro drug release was observed for 21 days and it inhibited *Staphylococcus* *aureus* growth.	[134]
Corn starch	Clonidine	Transdermal patches	Transdermal patches with a single dose of 30 μg hydrogel had an effect 15 min after application in treated mice.	[143]
Corn starch	Salicylic acid	Hydrogel membrane	The diffusion of the drug through the membrane was 4.11 × 10^−6^ cm^2^/s. The hydrogel was tested as an artificial skin for transferring nutrients or medicines, or for healing substances to the target area.	[144]
Corn starch/ethylene vinyl alcohol blend	Non-steroid anti-inflammatory agent	Drug delivery carriers	In vitro tests showed an immediate burst effect, followed by a slower, controlled release of the drug that lasted up to 10 days.	[145]
Potato starch	Ibuprofen, benzocaine, and sulphapyridine	Starch-based stable carriers	Encapsulation altered starch digestion; resistant starch was available in the colon for fermentation.	[146]
Glutinous rice starch, sodium alginate, and calcium chloride	Metformin hydrochloride	Hydrogel beads for controlled drug delivery	The initial drug entrapment efficiency was very low for the metformin hydrochloride because of its high solubility. Encapsulation improved it when combined with pre-gelatinized starch gel.	[147]
*Ensete ventricosum* starch	Epichlorohydrin	Drug-release sustaining pharmaceutical excipient	The in vitro drug release profile showed a minimum burst release, followed by a sustained release for 12 h.	[148]
Starch-clay composites	Tramadol	Tablet formulations	The controlled drug release of tramadol from starch-clay biocomposites was achieved in approximately 350 min.	[149]
Starch-chitosan	Hydroxyurea	Cancer therapy	The drug release was sensitive to pH and increased in the acid environment. The drug/starch/chitosan had a toxicity effect and, at certain concentrations, killed cancer cells.	[150]
PVA-corn starch hydrogel	Erythromycin	Wound dressing	The release of erythromycin from the PVA/corn starch network was higher than the drug containing PVA hydrogel (after 1800 min, released 76.7 mg of the total drug).	[151]
Corn starch-sponge matrix	Uranine, indomethacin, and nifedipine	Sustained-release capsule	After intraduodenal administration, 2.5% of the capsule exhibited a sustained release of the drug in the plasma.	[152]
Starch-poly-ε caprolactone	Dexamethasone	Drug delivery and tissue engineering applications	The drug from the outermost layer of the microparticles was quickly released. In vitro tests showed a sustained-release pattern for 30 days.	[153]
High-amylose starch-microcrystalline cellulose	Ranitidine hydrochloride	Gastric-floating drug delivery systems	In vitro tests indicated that the system with 3:7 (wt./wt.) starch/cellulose ratio maintained the buoyancy for more than a day; the drug release was 45.87% in the first hour, followed by a sustained release for up to 10 h.	[154]
Maize starch	Probiotics, e.g., *Lactobacillus plantarum*	Microencapsulated probiotic	In low acid environments, *L. plantarum* encapsulated in the starch matrix was more stable. After simulated digestion and heating treatments, the cells maintained their high viability, unlike formulations with native starch	[155]

**Table 5:** Utilization of alginate as a probiotic encapsulating material.

Biopolymers	Encapsulated Strain	Encapsulation Method	Benefits	References
Alginate and gelatin	*Lactobacillus rhamnosus*	Extrusion	The cells of *L. rhamnosus* survived in beads with 10^5^ CFU/g after four months (initially 10^9^ CFU/g).	[191]
Alginate	*Lactococcus lactis* spp. *cremoris*	Extrusion	No release of bacteria in the stomach simulated condition (first 120 min) or the survival in the intestinal fluid until 240 min.	[192]
Alginate	*Bifidobacterium pseudocatenulatum*	Extrusion	None of the uncoated probiotic cells survived after immersion in the simulated small intestine fluid. By contrast, 5.6 log10 CFU/g of viable probiotic cells remained in the tested microgels.	[193]
Alginate	*Staphylococcus succinus* and *Enterococcus fecium*	Extrusion	The encapsulated cells showed 98.75–88.75% of viability in simulated gastric fluids. Survival was constant throughout the storage time and decreased from 8.1 log CFU/mL to 7.9 log CFU/mL after 30 days of storage at 4 °C.	[194]
Alginate and milk	*Lactobacillus bulgaricus*	Extrusion	The viability of the encapsulated probiotic was the same after 120 min of incubation in an acid medium (simulated gastric fluid with pH 2.5). The viability of encapsulated *L. bulgaricus* was kept at 8 log CFU/g after 120 min of incubation at pH 2.0. Stability of the encapsulated probiotic can be preserved for one month after storage at 4 °C.	[195]
Alginate and starch	*Lactobacillus fermentum*	Lyophilization	The survival rate of the probiotic was significantly higher for microparticles blended with starch than those with no starch.	[196]
Alginate, chitosan, and locust beam	*Lactobacillus rhamnosus*	Freeze-drying	In contrast to the alginate-based capsules, the alginate locust beam capsules improved stress tolerance (6× for freeze-drying, 100× for thermotolerance, and 10× for acid).	[197]
Alginate and chitosan	*Saccharomyces cerevisiae Y235*	Emulsification	The viable microencapsulated cells were kept at 7.00 log CFU/g after six months at −20 °C and remained 6.29 log CFU/g after incubation in SGF for 2 h and in SIF for 12 h, reaching the standard value (10^6^–10^7^ CFU/g).	[198]
Alginate and chitosan	*Bifidobacterium pseudocatenulatum*		The highest stability of *B. pseudocatenulatum* was at the highest concentrations of alginate (4.41 g/100 mL) and chitosan (0.56 g/100 mL). Resistance of alginate–chitosan capsule in SGF was better than in SIF.	[199]
Alginate and chitosan	*Bifidobacterium breve*	Layer-by-layer	Three-layer coated matrix was the best method to increase viability from <3 log CFU/mL, seen in encapsulated cells, up to a maximum of 8.84 ± 0.17 log CFU/mL upon exposure to in vitro gastric conditions. Multilayer-coated alginate released their loads to the intestine with a gradual delivery over 240 min.	[200]
Alginate, starch, and chitosan	*Lactobacillus acidophilus*	Extrusion	Biopolymers ensured better stability of probiotics after exposure to SGF and SIF with 6.35 log CFU/g, while lower counts were noticed for freeze-dried microcapsules. During storage, cell viability of the probiotics stored in the freeze-dried form was up to six logs for 30 days and 135 days in the moist form when kept at room temperature.	[201]
Alginate, chitosan, and xanthan gum	*Lactobacillus plantarum*	Extrusion	Sequential incubation of biopolymers in SGF and SIF facilitated high survival of *L. plantarum* (95%) at pH < 2. Encapsulation improved storage stability of *L. plantarum* at 4 °C.	[202]

Abbreviations: CFU/g, colony-forming unit per gram; CFU/mL, colony-forming unit per milliliter; SGF, simulated gastric fluids; and SIF, simulated intestinal fluids.

**Table 6:** Chitosan use for probiotic encapsulation.

Biopolymers	Encapsulated Strain	Encapsulation Method	Benefits	References
Chitosan, agar, and gelatin	*Lactobacillus plantarum*	Emulsification	Particles with a diameter of approximately 6 mm did not solubilize in SGF 20 min after exposure. Cell viability in the biopolymer-free formula decreased completely after 2 h, unlike coated particles whose viability was 9.2 CFU/g after 2 h.	[206]
Chitosan and alginate	vaccine with *Lactobacillus plantarum*	Extrusion	The oral vaccine containing *L. plantarum*, used against spring viremia of carp virus, was effective even after 56 days due to the encapsulation.	[207]
Chitosan and xanthan gum	*Pediococcus acidilactici*	Extrusion	The encapsulated cells maintained their cell viability for 8 h in the gastrointestinal fluid with maximum release occurring after 24 h. The encapsulated cells maintained their viability for three days when tested in deionized water.	[208]
Chitosan and alginate	*Bifidobacterium breve*	Extrusion	In an acidic medium (pH 2), cell viability was maintained for 1 h. As pH increased (4 and 5), cell viability increased to 120 min. After 2 h, the swelling ratio decreased, a sign that the microcapsules began to disintegrate. Chitosan maintained cellular stability at pH 4 and 5, and alginate at pH 2.	[209]
Chitosan and alginate	*Lactobacillus reuteri DSM 17938*	Vibration technology	Unencapsulated cells were more labile to gastrointestinal stress conditions (reduction by 2.09 log cycles after 3 h). The encapsulated ones resisted better with a reduction of 0.82 log cycles.	[210]
Chitosan and alginate	*Saccharomyces boulardii*	Extrusion	Encapsulation of strains with chitosan and alginate facilitated maintenance of cell viability up to 6 h after administration in mice.	[211]
Chitosan and alginate	*L. acidophilus and L. casei*	Extrusion	Galactooligosaccharides potentiated the effect of microencapsulation. Cell viability was reduced by 3.1 logs for *L. acidophilus* and 2.9 logs for *L. casei* when tested at a very low pH (1.55) of SIF.	[212]
Chitosan and alginate	*Bacteria strain 4.1.Z (B. amyloliquefaciens, B. subtilis,* and *B. methylotrophicus)*	Vibration and extrusion	After lyophilization, the microcapsules maintained their viability (10^6^–10^7^ CFU/g) for about two months under refrigeration. Chitosan maintained the integrity of capsules for 24 h.	[213]
Chitosan and alginate	*Lactobacillus reuteri KUB-AC5*	Emulsification	The viability of non-encapsulated cells decreased in 40 min from 8 logs CFU/mL to <4 log CFU/mL, being completely eliminated after 1 h. The encapsulated cells were much more stable with a reduction of 1 log CFU/mL after 180 min at pH 1.8.	[214]
Chitosan and hydrochloride-alginate	*Bacillus licheniformis*	Orifice-polymerization method	The chitosan coating protected the microcapsules; cell release (6.19 CFU/mL) in 1 h in SGF (pH 2) and 4 h in the simulated intestinal fluid (pH 6).	[215]

Abbreviations: CFU/g, colony-forming unit per gram; CFU/mL, colony-forming unit per milliliter; SGF, simulated gastric fluids; and SIF, simulated intestinal fluids.

**Table 7:** Starch as an encapsulation material for probiotics.

Biopolymers	Encapsulated Strain	Encapsulation Method	Benefits	References
Rice starch	*Lactobacillus casei, Lactobacillus brevis,* and *Lactobacillus plantarum*	Extrusion	The viability of encapsulated cells (8.27/8.46/7.65 log CFU/g) was kept constant for two months at refrigeration. In contrast, non-encapsulated cells lost their viability by approximately 3 log CFU/g during storage.	[219]
Starch and pectin	*Lactobacillus plantarum*	Extrusion	Cell viability was reduced from 10 log CFU/g to 1 log CFU/g for free cells maintained for 2 h in gastric conditions (pH 1.5–3). Cells encapsulated in pectin had higher viability (4.6 log CFU/g) but the best protection was observed with the addition of starch to which the viability increased to 6.94 log CFU/g.	[220]
Starch from corn and rice	*Lactobacillus plantarum*	Freeze-drying	Encapsulated cells showed thermal stability and maintained their integrity for 35 min at 55 °C. Unencapsulated cells subjected to the same treatment lost their viability by 63% after only 10 min of exposure to 55 °C.	[221]
Starch, alginate, chitosan, and inulin	*Lactobacillus casei* and *Bifidobacterium bifidum*	Emulsification	Encapsulated *L. casei* and *B. bifidum* lost their viability when subjected to simulated gastric conditions for 120 min. Cell viability decreased from 25.10 × 10^10^ CFU/mL to 6.30 × 10^6^ CFU/mL for *L. casei*. Encapsulated *B. bifidum* lost 4.65 log/mL of the bacterial culture, while the unencapsulated form had undetectable cell viability after 90 min.	[222]
Starch and alginate	*Lactobacillus fermentum*	Emulsification	Encapsulated in the matrix, cells maintained viability when stored at 4 °C for 45 days. In environmental conditions, however, cells showed a decrease of 1.7 log after 24 h, with complete loss after 2 weeks.	[196]
Starch	*Lactobacillus paracasei*	Electrospinning	Tested at different storage temperatures (4, 25, and 37 °C), *L. paracasei* cells maintained their initial viability of 13.6 × 10 CFU/mL when stored for three weeks at 4 °C and 25 °C but not at 37 °C. Unencapsulated cells lost about 90% of their viability regardless of the storage temperature.	[223]
Maize starch, maltodextrin, and gum arabic	*Lactobacillus acidophilus*	Spray-drying	After 30days of storage at room temperature, only strains encapsulated with maltodextrin, namely gum arabic, maintained their cell viability of 10^6^ CFU/g. After 60 days, no encapsulating material prevented the loss of cell viability. Of the tested coatings, starch least protected the bacterial strains.	[224]
Taro and rice starch	*Lactobacillus paracasei*	Spray-drying	When stored, the taro–starch encapsulated strains were more stable; cells maintained their viability for a month, both at temperatures of 4 °C and 25 °C.	[218]
Cassava starch and alginate	*Lactobacillus brevis*	Emulsification	Encapsulation efficiency was higher than 89%. In gastrointestinal conditions, cell viability was better for microcapsules than free cells (96.07% compared to 76.51%). After 5 h of maintenance in the same conditions, viability of *L. brevis* encapsulated cells was 8.69 log CFU/mL, unlike the non-encapsulated ones with 6.87 log CFU/mL.	[225]
Starch and alginate	*Lactobacillus casei*	Extrusion	The addition of 2% starch to the alginate-based film-forming solution increased cell viability from 4 × 10^8^ to 3.1 × 10^11^. Increasing starch did not change the results. Tested under simulated gastrointestinal conditions, cell viability was maintained for up to 6 h.	[226]

**Table 8:** Cellulose as a probiotic encapsulating material.

Biopolymers	Encapsulated Strain	Encapsulation Method	Benefits	References
CMC and gelatin	*Lactobacillus rhamnosus*	Emulsification	After 120 min of exposure to SGF and SIF, cell viability was maintained at approximately 77.5% (4 log CFU/mL) in capsules and 60% (5 log CFU/mL) in free cells.	[228]
CMC and κ-carrageenan	*Lactobacillus plantarum*	Extrusion	Cellular stability was greatly improved for encapsulated samples: in an acidic medium (pH 2), it decreased from 10 log CFU/g to 0 after 90 min (non-encapsulated cells) and to about 8 log CFU/g after 120 min (encapsulated cells). During storage for 30 days at 4 °C, cell stability changed from 10 log CFU/g to 2 log CFU/g (free cells) and from 10 log CFU/g to 7 log CFU/g (encapsulated cells).	[229]
Cellulose and pectin	lactic acid bacteria	High-pressure microfluidization	Viability of non-encapsulated cells decreased from 9.56 to 5.29 log CFU/mL in an acid medium, while encapsulation protected cells (decrease of 1.88 log CFU/mL after 2 h in the same conditions).	[230]
CMC and inulin	*Lactobacillus plantarum*	Casting	Cell viability decreased during storage whether or not probiotics were encapsulated.	[227]
CMC and rice bran	*Lactobacillus reuteri*	Emulsification	After heat exposure (85 °C, 25 s), cell viability decreased by more than 57%, although *L. reuteri* is a thermotolerant bacterium. However, the survival rate of encapsulated cells was approximately 6 log CFU/g.	[231]
CMC and chitosan	*Lactobacillus rhamnosus*	Extrusion	Microencapsulated strains were stable at pH 2–4; at the highest pH value tested (12.5), all microcapsules disintegrated.	[232]
Cellulose, alginate, starch, and lecithin	*Lactobacillus rhamnosus*	Extrusion	Under gastric conditions, viability of encapsulated cells was 37% higher than that of free ones. Encapsulation had a positive effect on storage, in which viability decreased by 1.23 log (25 degrees) and 1.08 log (4 degrees), unlike free cells in which stability decreased by 3.17 and 1.93.	[233]
Cellulose and alginate	*Lactobacillus plantarum*	Extrusion and lyophilization	Lyophilized encapsulated cells showed the best stability in the simulated gastrointestinal conditions: gradual release of 2.6 × 10^6^ CFU/mL for 210 min. When refrigerated, encapsulated cells maintained viability for up to 160 days.	[155]
Cellulose and alginate	*Lactobacillus plantarum*	Extrusion-dripping	After 120 min in SGF, viability of non-encapsulated cells decreased by 66.6%, while encapsulated strains had a 58.4% better viability than that of free cells. The addition of cellulose protected the capsules from the action of pH.	[234]

Abbreviations: CMC, carboxymethyl cellulose; SGF, simulated gastric fluids; SIF, simulated intestinal fluids; CFU/g, colony-forming unit per gram; and CFU/mL, colony-forming unit per milliliter.

## Data Availability

The data presented in this study are available upon request from the corresponding author.
